# Reintervention for self-expandable metal stent dysfunction caused by tumor ingrowth using endoscopic holmium laser ablation

**DOI:** 10.1055/a-2650-9610

**Published:** 2025-07-29

**Authors:** Takeshi Ogura, Jun Matsuno, Takafumi Kanadani, Junichi Nakamura, Hiroki Nishikawa

**Affiliations:** 1 38588 Endoscopy Center, Osaka Medical and Pharmaceutical University Hospital, Osaka, Japan; 2 13010 2nd Department of Internal Medicine, Osaka Medical and Pharmaceutical University, Osaka, Japan

In cases of an occluded uncovered self-expandable metal stent (UCSEMS), reintervention is sometimes performed deploying additional UCSEMSs. However, re-intervention for an occluded UCSEMS due to tumor ingrowth might sometimes be challenging. Moreover, this procedure might be more difficult for patients who have undergone multiple SEMS deployments using the stent-in-stent technique. However, because of patients’ longer survival due to improvements in systemic chemotherapy such as immune checkpoint inhibitors, the frequency of reintervention may be increased. Endobiliary radiofrequency ablation may be one of the options to treat tumor ingrowth 1 2 , although long-term outcomes are still unclear. Compared with endobiliary radiofrequency ablation, an endoscopic holmium laser procedure may be safely performed because of direct visualization by cholangioscopy. A case of successful reintervention after multiple UCSEMS deployments by endoscopic holmium laser ablation is described.

A 92-year-old man previously underwent multiple UCSEMS deployments for cholangiocarcinoma. He also underwent seven reintervention sessions. He developed a stent obstruction, and therefore re-intervention was attempted ( Fig. 1 ). Guidewire insertion into the biliary tract was successfully performed, followed by cholangioscope insertion. On cholangioscopy, tumor ingrowth was observed ( Fig. 2 ). Because the cholangioscope could not be advanced into the hepatic hilar site due to tumor ingrowth, tumor ablation using a holmium laser was attempted ( Fig. 3 ). After tumor ablation, the tumor disappeared ( Fig. 4 ), and the cholangioscope could be advanced into the right hepatic bile duct. However, tumor ingrowth was also observed at this site. Tumor ablation using the holmium laser was again attempted, and the tumor was successfully ablated without any adverse events. Cholangiography showed no stricture ( Fig. 5 , Video 1 ). Therefore, additional stent deployment was not performed. Stent patency was obtained for four months until the patient’s death.

**Fig. 1:**
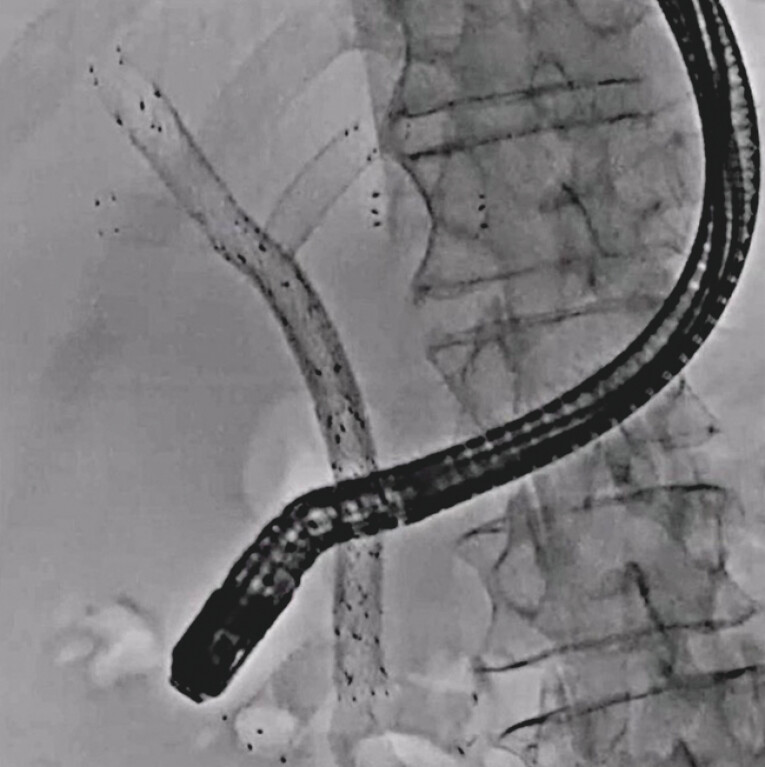
Deployment of multiple uncovered self-expandable metal stents.

**Fig. 2:**
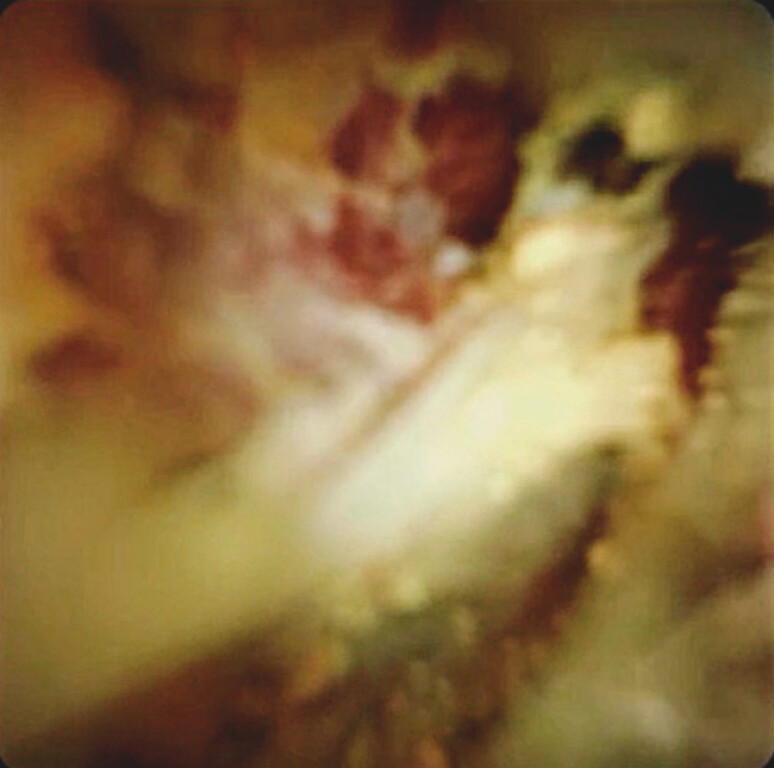
On cholangioscopy, tumor ingrowth is observed.

**Fig. 3:**
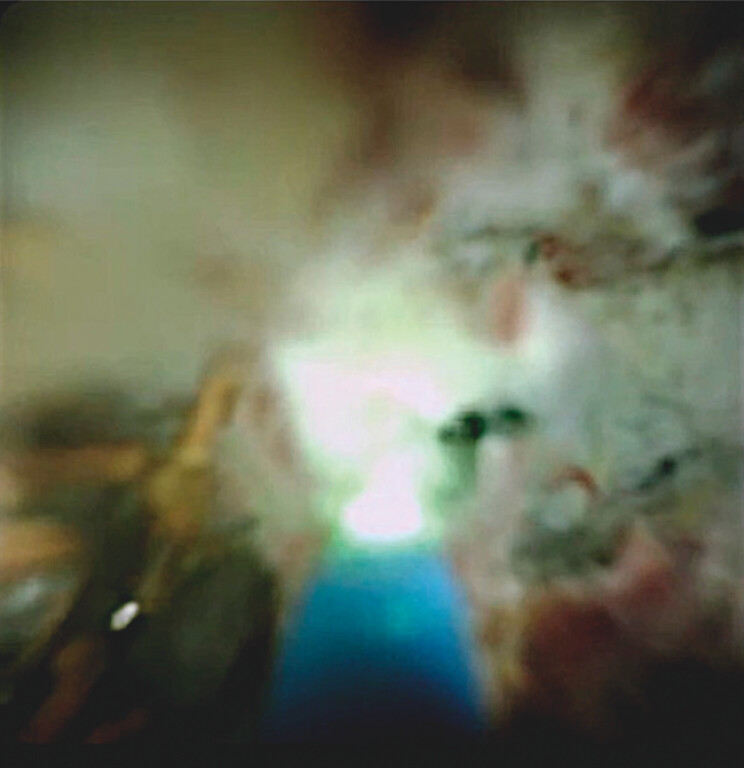
Tumor ablation using a holmium laser is attempted.

**Fig. 4:**
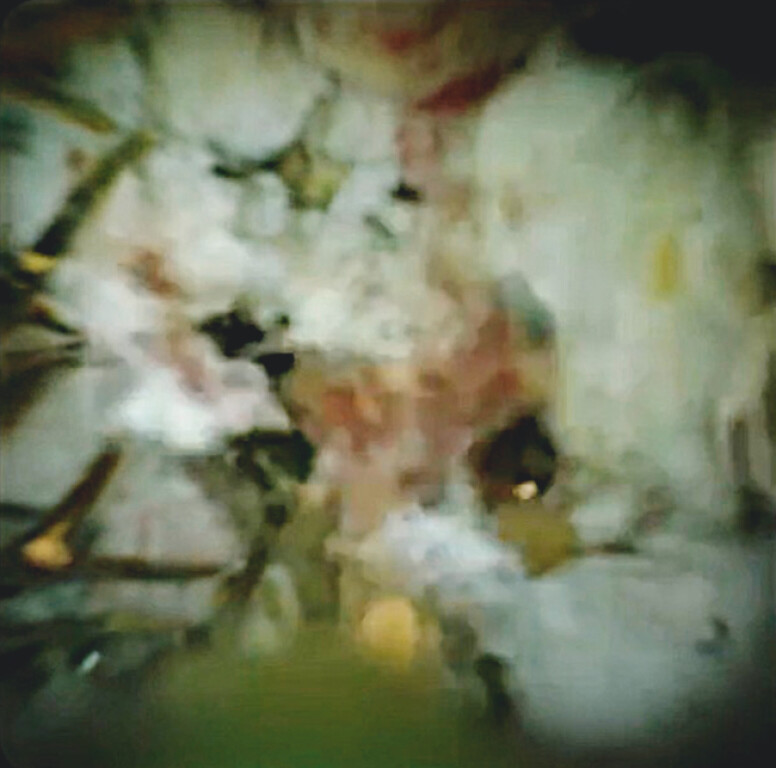
After tumor ablation, the tumor has disappeared.

**Fig. 5:**
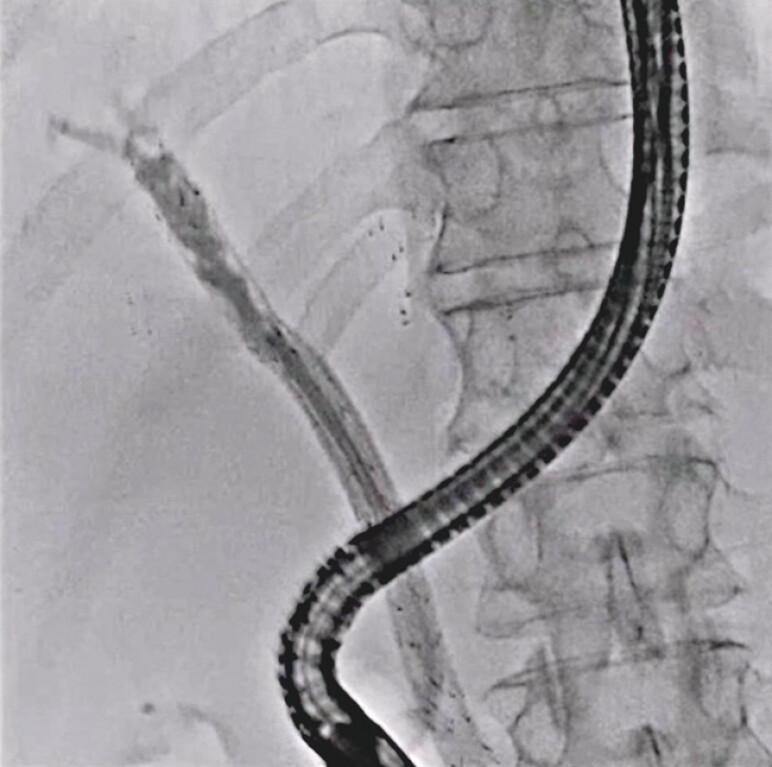
Cholangiography shows no stricture.

Reintervention for self-expandable metal stent dysfunction caused by tumor ingrowth using endoscopic holmium laser ablation.Video 1

In conclusion, tumor ablation using a holmium laser can be one of the options as a reintervention technique for an occluded UCSEMS.

Endoscopy_UCTN_Code_TTT_1AR_2AZ

## Notes

Endoscopy E-Videos https://eref.thieme.de/e-videos
*E-Videos* is an open access online section of the journal *Endoscopy* , reporting on interesting cases and new techniques in gastroenterological endoscopy. All papers include a high-quality video and are published with a Creative Commons CC-BY license. Endoscopy E-Videos qualify for HINARI discounts and waivers and eligibility is automatically checked during the submission process. We grant 100% waivers to articles whose corresponding authors are based in Group A countries and 50% waivers to those who are based in Group B countries as classified by Research4Life (see: https://www.research4life.org/access/eligibility/ ). This section has its own submission website at https://mc.manuscriptcentral.com/e-videos .
